# Atomistic Origins of Various Luminescent Centers and n-Type Conductivity in GaN: Exploring the Point Defects Induced by Cr, Mn, and O through an *Ab Initio* Thermodynamic Approach

**DOI:** 10.1021/acs.chemmater.4c00178

**Published:** 2024-06-19

**Authors:** Kamil Czelej, Mubashir Mansoor, Mehmet Ali Sarsil, Mert Tas, Yahya A. Sorkhe, Mehya Mansoor, Maryam Mansoor, Bora Derin, Onur Ergen, Servet Timur, Mustafa Ürgen

**Affiliations:** † Faculty of Chemical and Process Engineering, Warsaw University of Technology, Waryńskiego 1, 00-645 Warsaw, Poland; ‡ Department of Complex System Modeling, Institute of Theoretical Physics, Faculty of Physics, University of Warsaw, Ludwika Pasteura 5, 02-093 Warsaw, Poland; § Metallurgical and Materials Engineering Department, Istanbul Technical University, 34469 Maslak, Istanbul, Turkey; ∥ Department of Applied Physics, Istanbul Technical University, 34469 Maslak, Istanbul, Turkey; ⊥ Department of Electronics and Communications Engineering, Istanbul Technical University, 34469 Maslak, Istanbul, Turkey; # Department of Geological Engineering, Istanbul Technical University, 34469 Maslak, Istanbul, Turkey; ∇ Nuclear Engineering Department, Energy Institute, Istanbul Technical University, 34469 Maslak, Istanbul, Turkey; ○ Department of Mining Engineering, Istanbul Technical University, 34469 Maslak, Istanbul, Turkey

## Abstract

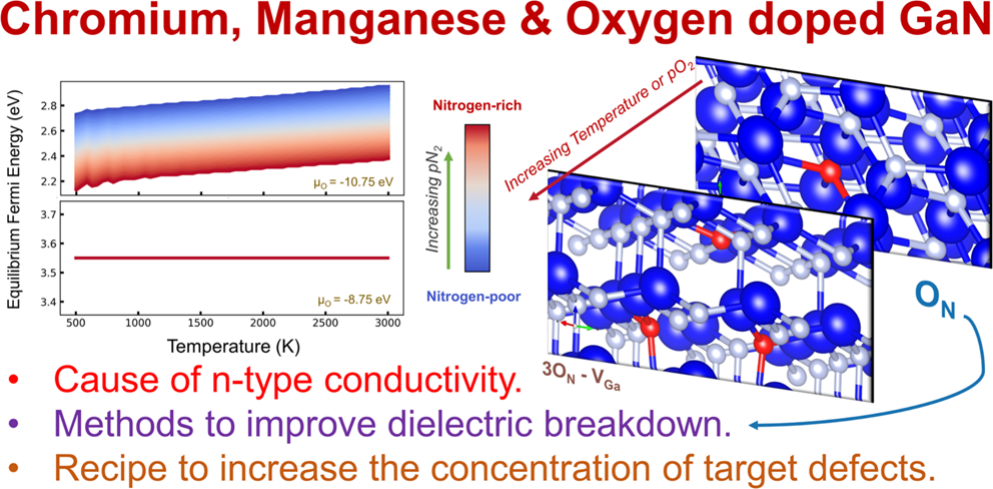

GaN is a technologically indispensable material for various optoelectronic properties, mainly due to the dopant-induced or native atomic-scale point defects that can create single photon emitters, a range of luminescence bands, and n- or p-type conductivities. Among the various dopants, chromium and manganese-induced defects have been of particular interest over the past few years, because some of them contribute to our present-day light-emitting diode (LED) and spintronic technologies. However, the nature of such atomistic centers in Cr and Mn-doped GaN is yet to be understood. A comprehensive defect thermodynamic analysis of Cr- and Mn-induced defects is essential for their engineering in GaN crystals because by mapping out the defect stabilities as a function of crystal growth parameters, we can maximize the concentration of the target point defects. We therefore investigate chromium and manganese-induced defects in GaN with *ab initio* methods using the highly accurate exchange–correlation hybrid functionals, and the phase transformations upon excess incorporation of these dopants using the CALPHAD method. We also investigate the impact of oxygen codoping that can be unintentionally incorporated during crystal growth. Our analysis sheds light on the atomistic cause of the unintentional n-type conductivity in GaN, being O_N_-related. In the case of Cr doping, the formation of Cr_Ga_ defects is the most dominant, with an *E*^+/0^ charge transition at *E*_VBM_ + 2.19 eV. Increasing nitrogen partial pressure tends to enhance the concentration of Cr_Ga_. However, in the case of doping with Mn, several different Mn-related centers can form depending on the growth conditions, with Mn_Ga_ being the most dominant. Mn_Ga_ possesses the *E*^2+/+^, *E*^+/0^, and *E*^0/–^ charge transitions at 0.56, 1.04, and 2.10 eV above the VBM. The incorporation of oxygen tends to cause the formation of the Mn_Ga_–V_Ga_ center, which explains a series of prior experimental observations in Mn-doped GaN. We provide a powerful tool for point defect engineering in wide band gap binary semiconductors that can be readily used to design optimal crystal growth protocols.

## 1 Introduction

Gallium nitride (GaN) belongs to a family of III–V compound wide band gap semiconductors. It typically stabilizes in the wurtzite phase under ambient conditions. GaN is a crucial material for a variety of technologically essential applications, including power electronics,^1^ blue- and white-light-emitting diodes (LEDs),^2^ lasers,^3^ solar cells,^4^ and photocatalysis.^5^ GaN is separated from other wide band gap semiconductors by their unique ability to be doped in n- and p-type sets.^6^ The functionality of GaN-based devices is heavily influenced by point defects.^7^ While these defects can be beneficial in some cases, such as in photocatalytic materials where they can act as active sites or as a potential quantum bit in quantum information science.^8,9^ Their presence can often negatively impact the performance, for instance, by nonradiative recombination in light emitters,^10^ making it crucial to understand the nature and behavior point defects that arise during growth or postprocessing steps. In particular, incorporating 3d transition metals such as chromium and manganese into the GaN lattice has raised significant interest in the optoelectronics research community. Mn and Cr-induced defects in GaN give rise to rich optical, electrical, and magnetic properties that can be used in spintronics, photonics, and, recently, quantum information processing.^11^ It has been demonstrated that the Curie temperature (*T*_c_) of Mn-doped GaN can be tuned by Mn concentration and reach as high as 945 K for 9% Mn.^12,13^ Hashimoto et al.^14^ utilized electron cyclotron resonance assisted molecular beam epitaxy (ECR–MBE) to grow GaN successfully: Cr on a sapphire substrate, achieving *T*_c_  ≥  400 K for a Cr concentration of 7%. Cr-doped GaN gives rise to a very sharp infrared emission at ZPL = 1.193 eV that originates from internal transition within the 3d shell of the Cr^4+^ ion.^15^ Koehl et al. incorporated Cr^4+^ into GaN and employed optically detected magnetic resonance spectroscopy (ODMR) to demonstrate coherent manipulation and control of the *S* = 1 electronic ground state.^16^ In addition, this emitter’s very weak electron–phonon coupling encourages further attempts to integrate optically active quantum states into widely used optoelectronic materials. Advancements in understanding the role of native defects, unintentional contaminants, and dopant impurities have played a significant role in the development of GaN. However, there are still some points to be clarified on the contribution of these defects to the properties of GaN. When Mn or Cr is doped into GaN during growth, a variety of point defects and their complexes with these transition metals can be destructive for target applications, which needs to be identified from an atomistic perspective. Additionally, the atomistic cause of a range of photoluminescence (PL) peaks observed in undoped GaN or doped GaN is still under debate, some of which have been attributed to various native defects.^17^ Similarly, the cause of unintentional n-type conductivity of GaN is attributed to nitrogen vacancies,^18^ while others believe it results from unwanted impurities.^19−22^

Given the wide variety of defects, charge states, and ionization levels, understanding the experimental spectroscopic and optoelectronic results relative to specific defect species is challenging and requires significant theoretical input. This complexity is because the defects in GaN can act as either donors, contributing electrons to the conduction band under certain conditions, or acceptors, which donate holes to the valence band. Notably, many defects can function as electron donors and acceptors, resulting in changes in their charge states and may induce deep donor and acceptor levels within the band gap. Accordingly, during doping native defects can act as compensation, passivation, and recombination centers. Thus, understanding the formation thermodynamics of the complex (dopant–native) defects and their electronic structure becomes crucial for elaborating impurity-induced optoelectronic properties.^23^ Therefore, it becomes possible to design a material of interest by computing the optoelectronic properties of a defect complex. However, knowing the processing conditions that can give rise to the defect of interest is equally vital because growth and postprocessing parameters influence the defect thermodynamics of a crystal and thereby change the defect stabilities in the system. Accordingly, a computational methodology that satisfies both requirements is needed.

Our comprehension of point defects in GaN has been primarily impacted by the advancements in the predictive capability of *ab initio* density functional theory (DFT) modeling.^24^ In the past, DFT calculations within the local density approximation (LDA) and generalized gradient approximation (GGA) had limitations due to the band gap problem. However, the use of hybrid functionals has enabled quantitative predictions of the thermodynamic transition levels, formation energies, and atomic structures of defects. Additionally, first-principles methods have been developed to consider the role of electron–phonon coupling, enabling the accurate calculation of optical transitions,^25^ nonradiative and radiative recombination rates,^26−28^ and thermal emission rates involving point defects.^29^ These advances have led to a better understanding of point defects in GaN and opened up new research avenues, such as investigating the defect behavior in alloys and the role of excited states of defects.

Various defects in GaN and other materials are frequently analyzed computationally through the formation energy–Fermi energy diagrams. However, from a process engineering perspective, these diagrams may not immediately provide helpful information. A more practical approach is using Kröger–Vink diagrams, which facilitate defect engineering.^30,31^ Kröger–Vink diagrams are commonly employed to demonstrate defect concentrations at constant temperature as a function of nitrogen and oxygen partial pressure for nitrides and oxides, respectively. By computing defect concentrations over a broad temperature range using a canonical ensemble and generating what we refer to as monolithic Kröger–Vink diagrams to display defect concentrations as a function of temperature and dopant chemical potential, it becomes feasible to optimize growth conditions and find the defect transformations based on growth parameters.

Here, we generate a series of monolithic Kröger–Vink diagrams for predicting the defect concentrations in GaN grown with either chromium or manganese chemistry, as determined by process parameters such as temperature, partial pressures of gases, and chemical potential. We also consider oxygen as a trace impurity and its complexes with native Cr- or Mn-containing defects. First, we conducted hybrid DFT calculations of each defect in different charge states to determine its total energy and charge transition levels and provide high-quality input data for solving the nonadiabatic thermodynamic equations for this system of dopants. Our study clearly identifies the cause of unintentional n-type conductivity in GaN, linked to O_N_-related defects. Controlling growth conditions can suppress this defect. In the case of Cr doping, the formation of the Cr_Ga_ defect is the most dominant, with an *E*^+/0^ charge transition at *E*_VBM_ + 2.19 eV. Increasing nitrogen partial pressure tends to enhance the concentration of Cr_Ga_. However, in the case of doping with Mn, several different Mn-related defects can form depending on the growth conditions, with Mn_Ga_ being the most dominant. Mn_Ga_ possesses *E*^2+/+^, *E*^+/0^, and *E*^0/–^ charge transitions at 0.56, 1.04, and 2.10 eV above the VBM. The incorporation of oxygen tends to cause the formation of Mn_Ga_–V_Ga_ defect with *E*^3+/2+^, *E*^2+/0^, and *E*^0/–^ charge transitions at 0.42, 1.42, and 1.80 eV above the VBM, respectively, which explains a series of prior experimental observations in Mn-doped GaN. This research provides a comprehensive thermodynamic blueprint for analyzing phase stability and defect equilibria in Cr, Mn, and O-doped GaN, addressing the topic from both process engineering and defect analysis perspectives. This inquiry promises to unveil critical discoveries in Cr- and Mn-doped GaN, contributing to the academic conversation with a combination of clarity and excitement.

## 2 Methods

### 2.1 Calculation of Phase Diagrams for the GaN Phase

The CALPHAD method is used to study the phase stability of GaN as a function of temperature, nitrogen, and oxygen partial pressure, which will provide the processing parameter limits that will be used along with the DFT calculations for a more comprehensive analysis. The impact of pO_2_ and pN_2_ is also taken into consideration, given that residual oxygen and nitrogen partial pressure in a growth chamber can have a substantial impact on the phase stability of GaN. To predict the phase stabilities, we performed thermochemical simulations through the Phase Diagram module of FactSage 8.2 by utilizing the SGTE 2022 and FactPS databases,^32^ and also used Thermo-Calc software 2023b, which facilitates a variety of thermodynamic and phase diagram calculations for equilibrium problems using the CALPHAD method.^33^ We employed the robust Gibbs Energy Minimizer of Thermo-Calc software to compute phase equilibria and thermodynamic properties for the specified systems of GaN–Cr, GaN–O, and GaN–Mn. The CALPHAD method involves deriving thermodynamic functions for a system based on all accessible experimental and computational databases, expressing these functions as polynomials of the chemical composition and process parameters. Subsequently, numerical optimization techniques are applied to determine the values of polynomial coefficients, which are explained in detail elsewhere.^34^

The phase equilibria were calculated as a function of the nitrogen partial pressure, dopant activity, and temperature for a selected composition of 5 mol % dopant mixture. For all cases, the total pressure was fixed at 1 atm, and the temperature range was chosen as 500–2700 K. The system size is 1 mol, and diagrams are calculated for the Dormant gas phase conditions. The used databases are the SSOL8 SGTE Solutions Database v.8, SSUB7 Substances Database v.6, and the TCSI1–TCS Ultrapure Silicon Database Version 1.2. We considered only the stable phases in this study.

### 2.2 Electronic and Atomic Structure Calculations for Defects in GaN

Our electronic structure calculations are based on DFT and the projector augmented wave (PAW) method as implemented in the Vienna Ab Initio Simulation Package (VASP).^35,36^ We employed the screened range-separated nonlocal hybrid functional of Heyd, Scuseria, and Ernzerhof (HSE).^37,38^ We set the fraction of exact exchange to α = 0.3 while the screening parameter is ω = 0.2 1/Å so that the calculated band gap of 3.55 eV is close to the reported experimental values 3.47–3.51 eV.^39−41^ The calculated lattice constants of GaN *a* = 3.193 Å and *c* = 5.186 Å perfectly agree with the reported experimental values *a* = 3.194 Å, *c* = 5.186 Å reported by Leszczynski et al.^42^

To minimize finite-size effects, we applied a large 300-atom (5 × 5 × 3) wurtzite supercell. This enables accurate sampling of the first Brillouin zone using the Γ point, allowing, in turn, inspection of Kohn–Sham wave functions with correct symmetry and degeneracy. Defects in the supercell were relaxed in constant volume until the Hellmann–Feynman forces acting on each atom dropped below 0.01 eV/Å (∼0.01 meV/atom). The plane-wave cutoff energy of 450 eV was applied during structural relaxation. Spin polarization was considered for each defect.

### 2.3 Formation Energies, Charge Transition Levels of Defects, Binding Energies, and Zero Phonon Lines (ZPLs)

We computed the formation energy Δ*H*_f_^q^ of a defect as a function of the electron chemical potential *E*_F_ (equilibrium Fermi energy) in the band gap using the standard formula eq 1.^43^
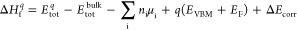
1Where the Fermi level *E*_F_ is referenced to the valence band maximum (VBM) of nondefective GaN, *q* denotes the charge state of a defect, *E*_tot_^q^ is the total energy of the supercell containing the defect, *E*_tot_^bulk^ is the total energy for the perfect crystal in the equivalent supercell, *n*_i_ indicates the number of atoms of type i (either host or impurity atoms) that were added to (*n*_i_ > 0) or removed from (*n*_i_ < 0) the supercell to create the defect, and μ_i_ is the chemical potential of the corresponding atoms (i = Ga, N, O, Cr, Mn). The chemical potential μ_N_ represents the energy of the reservoir with which nitrogen atoms are exchanged, reflecting the experimental conditions. It may vary between N-rich (Ga-poor) and N-poor (Ga-rich) extremes, with bounds set by the computed formation enthalpy of GaN: Δμ_Ga_ + Δμ_N_ = Δ*H*(GaN) = −1.11 eV. The last term Δ*E*_corr_ denotes the finite-size correction according to the Freysoldt correction scheme.^44^

The thermodynamic charge transition level *E*^*q*_1_/*q*_2_^ can be defined as the Fermi level position below which the defect is stable in charge state *q*_1_ and above which it is stable in charge state *q*_2_. It is calculated as shown in eq 2.
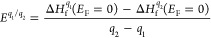
2

Zero phonon line (ZPL) associated with the electron or hole capture process is expressed as the energy difference between the conduction band minimum *E*_CBM_ and charge transition level *E*^*q*_1_/*q*_2_^ for electron capture, or the energy difference between charge transition level *E*^*q*_1_/*q*_2_^ and the valence band maximum *E*_VBM_ for hole capture^45,46^
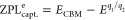
3
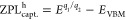
4in the case of complex defects, we calculated the binding energy defined as
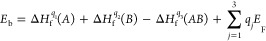
5where Δ*H*_f_^*q*_1_^(*A*) is the formation energy of component *A* in charge state *q*_1_ at Fermi level *E*_F_, Δ*H*_f_^*q*_2_^(*B*) is the formation energy of component *B* in charge state *q*_2_ at Fermi level *E*_F_, Δ*H*_f_^*q*_3_^(*AB*) is the formation energy of complex *AB* in charge state *q*_3_ at Fermi level *E*_F_. According to this definition, positive binding energy indicates a tendency toward cluster formation.^47,48^

### 2.4 Formulation for Kröger–Vink Diagrams and Defect Equilibria

Kröger–Vink diagrams are an effective way of demonstrating the defect concentrations as a function of process parameters. Such diagrams are based on equilibrium formation energies of defects, which depend on the equilibrium Fermi energy of the defect ensemble. The equilibrium Fermi energy and consequently the defect concentrations are achieved only through charge neutrality. The concentrations of defects are intertwined with the equilibrium Fermi energy, which strongly depends on the concentrations of all available charged defects in the solid, free electrons, and holes. The sum of all charged species (negative and positive) should equate to zero net charge for the entire system. Therefore, by solving for charge neutrality, it becomes possible to achieve equilibrium concentrations based on a given defect ensemble. The benefit of this canonical approach is that no bookkeeping is necessary for defect–defect reactions, and a more comprehensive overview of the defect equilibria is achieved. To compute the equilibrium Fermi energy (*E*_F_), the following eqs 6–8 should be solved in a self-consistent manner.^30,49^
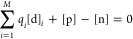
6

Equation 6 represents the charge neutrality condition, where [d] is the concentration of a defect *i* with charge *q*_*i*_. *M* is the total number of defects considered for a given defect ensemble. Hole and electron concentrations are given by [p] and [n], respectively, and are calculated as follows.
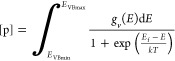
7
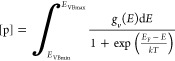
8Here, *g*_v_(*E*) and *g*_c_(*E*) represent the density of states for valence and conduction bands, respectively, as calculated by the HSE functional for the pristine GaN supercell. To calculate defect concentration [d] during synthesis and crystal growth, the nonadiabatic approach is taken into account when considering the chemical potentials of dopants (O, Mn, Cr), Ga and N. In this approach, the chemical reservoir (μ_i_) of the relevant species in the surrounding environment is considered. However, given that GaN is a binary compound, the nitrogen chemical potential is linked to the Ga chemical potential, as shown in eq 9.
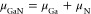
9When considering the equilibration of a dopant atom’s chemical potential with respect to chemical species in the surrounding environment, as a function of the constituent’s thermodynamic activity and temperature, we have considered the chemical potentials of the reference molecules and solids (Ga, N_2_, Cr, Mn, O_2_) as implemented before,^30,49,50^ and as variables.
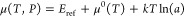
10

The *E*_ref_ term is the DFT calculated energy of the reference atom described above, using the HSE functional, and μ^0^ is the temperature-dependent change in chemical potential that has been obtained from the JANAF database.^51^ The last term accounts for the thermodynamic activities, and for gases such as N_2_, it is simply the partial pressure of the gas. Substituting the chemical potentials achieved into μ_i_, and considering defect concentrations [d], a self-consistent solution to the equilibrium Fermi energy and defect concentrations is achieved.
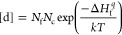
11

The Δ*H*_f_^*q*^ term for each defect of charge *q* is retrieved from eq 1. *N*_f_ represents the number of possible defect configurations of the same energy, and *N*_c_ is the number of maximum possible sites for the defect, and we have taken the *N*_f_*N*_c_ factor as a constant based on the GaN structure, as the variations in this factor for each defect does not imply any noticeable change in the final outcomes of the KV plots. In short, the temperature-dependent concentrations are calculated by finding the temperature-dependent equilibrium Fermi Energy and defect formation energies. This is achieved through the primary consideration of charge neutrality and the finite temperature effects are captured through changes in the chemical potentials of defect constituents, as it was carried out by Van de Walle,^50,52^ and a recent study by Somjit and Yildiz.^53^

## 3 Results and Discussion

### 3.1 Thermodynamic Phase Stability of GaN and Phase Transformations

**Figure 1:**
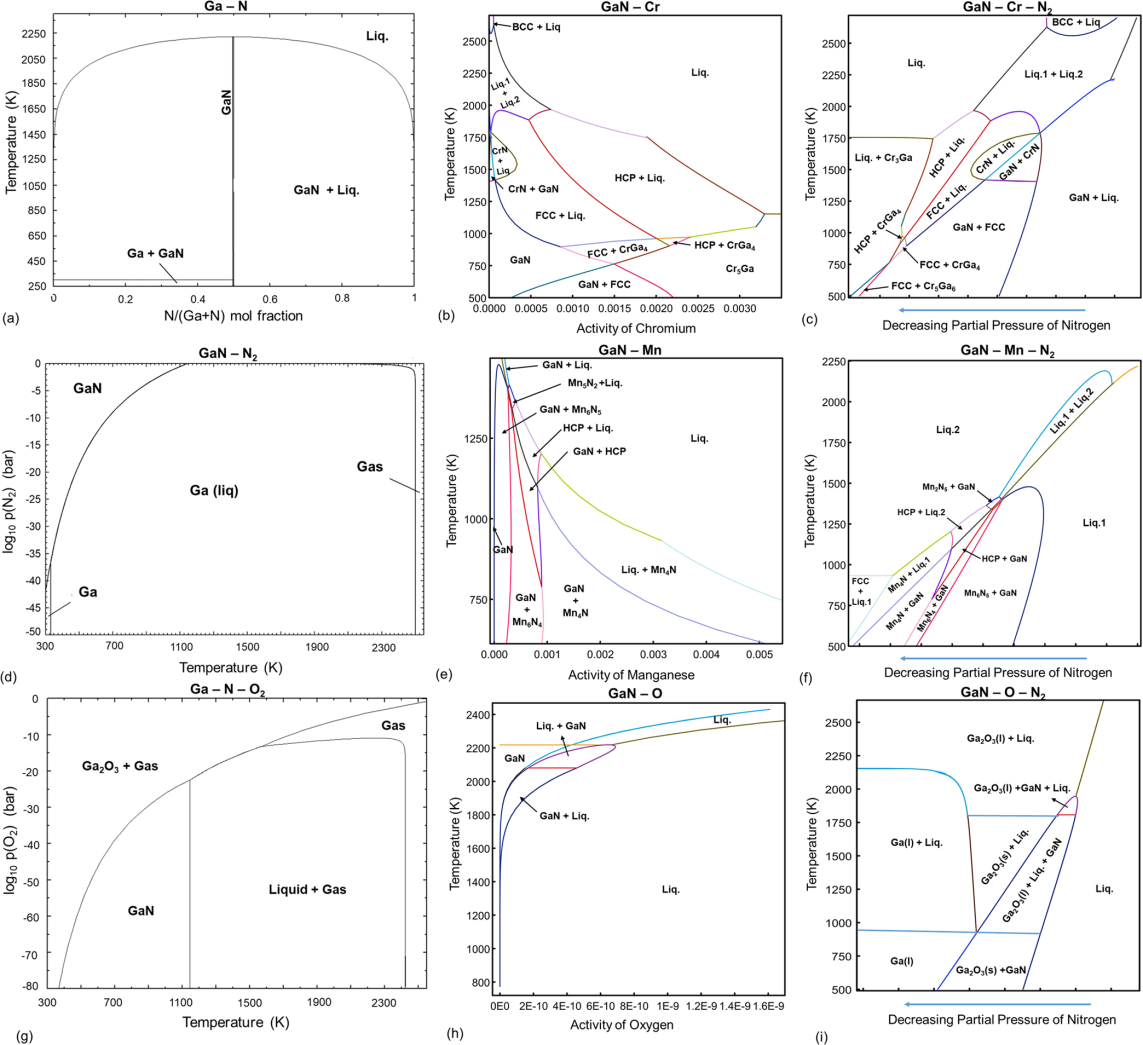
Phase diagrams of Ga–N as a function of molar stoichiometries (a) and the phase stability diagrams of GaN–Cr (b), GaN–Cr–N_2_ (c), GaN–N_2_ (d), GaN–Mn (e), GaN–Mn–N_2_ (f), Ga–N–O_2_ (g), GaN–O (h), and GaN–O–N_2_ (i) are shown as a function of dopant activity or nitrogen partial pressure. The wurtzite GaN structure is stable only under extremely low pO_2_ values of less than 10^–25^ atm at 1000 K or less. Higher temperatures promote phase decomposition in the presence of the slightest residual oxygen or nitrogen deficiency. Similarly, the phase transformations in the case of excess Cr, O, and Mn dopant activities are illustrated. The FactSage thermochemical databases are used for the calculations shown in (a, d, g). The remaining plots are calculated through Thermo-Calc thermochemical databases by considering a system size of 1 mol with 0.95 mol of GaN and 0.05 mol of dopant.

The Ga–N phase diagram produced with the CALPHAD method shows a wurtzite-structured line compound of GaN at 50 atomic percent of Ga and N, with a congruent melting point of approximately 2200 K, (Figure 1a). Our calculation results are close to the values found in the literature on the melting/decomposition point of GaN that were reported by Porowski et al.,^54^ Harafuji,^55^ and Piechota^56^ which indicate a melting point of approximately 2500 K, based on molecular dynamics simulation, with the study by Piechotra et al. indicating a spread of nearly 1000 K on the melt evolution. However, the CALPHAD-based thermochemical databases on FactSage and Thermo-Calc produce the same congruent melting, as seen in Figure 1a. Moreover, the stability of GaN is strongly dependent on the nitrogen partial pressure (Figure 1d). At temperatures above 1300 K, a pN_2_ of 1 atm or higher is required to avoid dissociating the GaN phase into liquid Ga and N_2_ gas. At lower temperatures, the thermodynamic stability increases; therefore, it is possible to reduce the pN_2_ even further with decreasing temperature (nitrogen-poor conditions).

The Thermo-Calc thermochemical databases have been used to better understand the phase stability region of GaN in the presence of oxygen, chromium, and manganese. The plots in Figure 1b–i (except for (d, g)) demonstrate the phase equilibria for a constant input composition of 0.95 mol of GaN and 0.05 mol of the dopant, with the system size being 1 mol. Given that solid solution databases are not comprehensive enough for such dopants in GaN, our *ab initio* results in the following sections provide a clearer picture. However, this thermochemical study aims to elucidate the phase transformations better once the solution is saturated and the solubility limits have been reached. Nevertheless, Cr is relatively more soluble than Mn and O, with the solubility of Cr being highest at temperatures below 1000 K (Figure 1b), while the oxygen solubility is highest at temperatures close to the melting point of GaN at approximately 2200 K. However, phase decomposition of the GaN is seen upon an increase in the thermodynamic activity of oxygen (Figure 1h), similar to the estimations through the FactSage databases (Figure 1g).

Upon increased activity of Cr, through increased temperature, Cr concentration, or otherwise, the formation of cubic FCC-structured GaN, and eventually CrN is inevitable. The transformation to cubic GaN is experimentally seen by Shanti et al.^57^ upon excess Cr doping. Further, increased Cr activity causes the evolution of two immiscible liquid phases (Liq. 1 and Liq. 2), as seen in Figure 1b,c. The FCC-structured CrN transforms into HCP-structured Cr_2_N in the case of a further increase in the Cr activity and a decrease in the nitrogen partial pressure. The phases indicated as FCC and HCP demonstrate chromium nitrides with limited solubility of Ga as well. These CALPHAD findings perfectly agree with the transmission electron microscopy (TEM) experimental observations on Cr–N thin films reported by Li et al.,^58^ where the formation dynamics of CrN and Cr_2_N are consistent with our computational findings reported here. In case of extreme N_2_ deficiency or increased Cr activity, the evolution of FCC CrN or HCP Cr_2_N becomes unlikely, and metallic alloys of Cr–Ga form, such as CrGa_4_ and Cr_5_Ga, which is also consistent with prior studies on Cr–Ga alloys.^59,60^

In the presence of Mn, beyond the solubility limit of Mn in GaN, several manganese nitrides can form, namely, Mn_6_N_5_, Mn_6_N_4_, Mn_5_N_2_, and Mn_4_N, with decreasing nitrogen molar ratio as the manganese activity increases or the nitrogen partial pressure decreases, consistent with prior experimental findings.^61,62^ The emergence of immiscible liquid phases is seen in the case of Mn addition, with the total liquidus temperature being highly dependent on the pN_2_ and Mn activity, as seen in Figure 1e,f, similar to the case of the addition of O and Cr addition.

In the presence of even the slightest oxygen concentrations, Ga_2_O_3_ and liquid phases are formed, followed by metallic Ga formation under extremely low pN_2_. In comparison, GaN can be stable in the presence of Mn and Cr with dopant activities that are orders of magnitude higher (Figure 1g–i). Based on these thermodynamic data, it is rather difficult to avoid or eliminate oxygen doping in GaN from a process engineering perspective. In the presence of residual oxygen, the stability of GaN is further diminished. Under oxygen-rich conditions, the formation of Ga_2_O_3_ becomes inevitable (Figure 1g).

**Figure 2:**
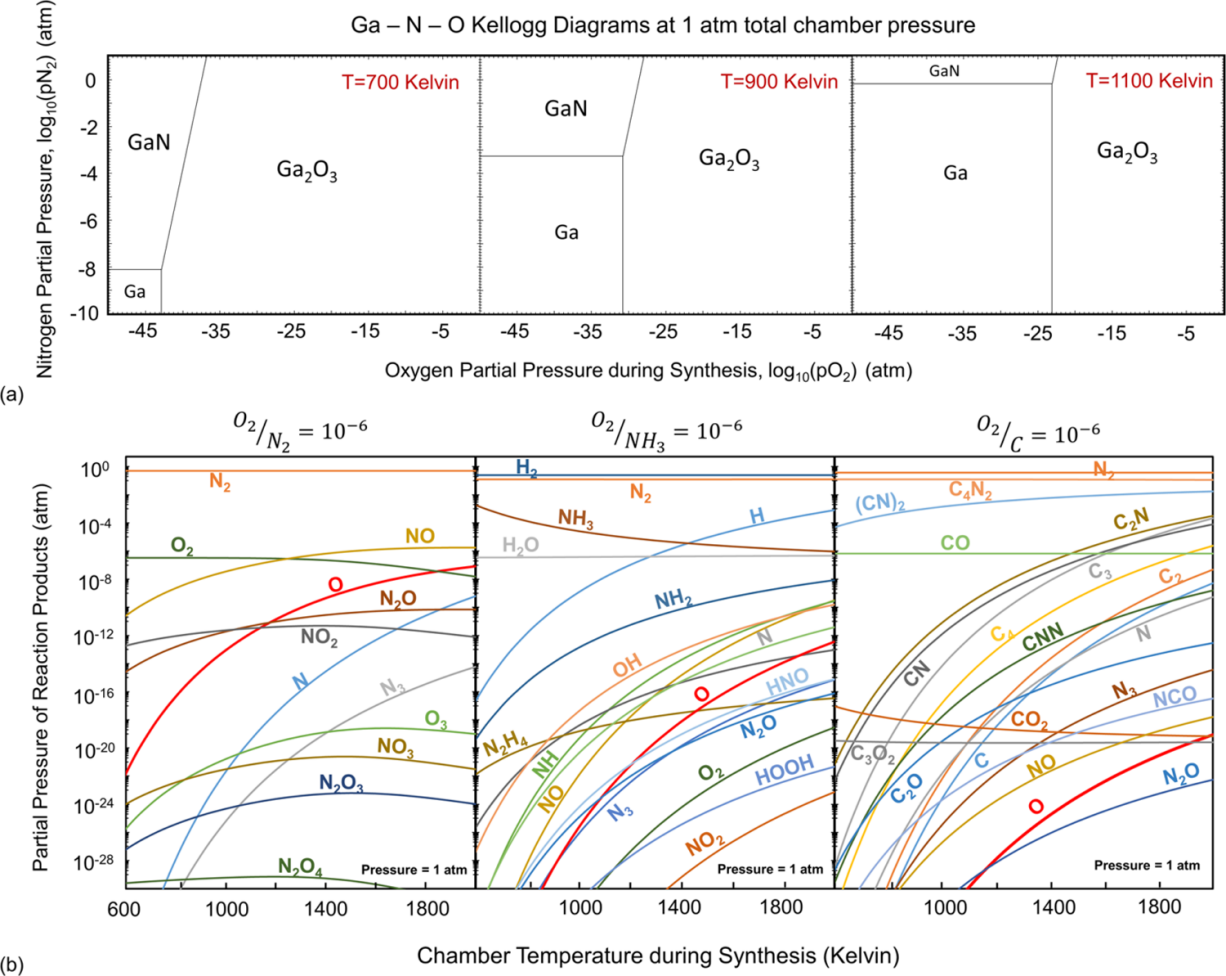
Kellogg diagrams of the Ga–N–O system are plotted for 700, 900, and 1000 K under 1 atm of total chamber pressure, based on the pO_2_ and pN_2_, demonstrating the changes in the GaN stability (a). The gaseous reaction products are also shown for the cases where 1 ppm of residual oxygen is exposed to nitrogen only and ammonia and carbon-containing mixture, which demonstrate in the presence of hydrogen and carbon, the equilibrium oxygen content is excruciatingly low, well under the levels necessary for GaN synthesis at temperatures close to 1000 K.

Based on the CALPHAD data, the maximum pO_2_ acceptable during growth is 10^–25^ atm at the growth temperature of 1000 K. Under oxygen-free conditions, GaN is stable up to 2200 K. However, in the presence of even 10^–25^ atm of residual oxygen partial pressure, the GaN phase dissociates at nearly 1100 K. The thermochemical results shown in Figure 1 explain why the numerous GaN growth systems operate at a temperature range of 1000–1300 K.^63^ To better understand the impact of residual oxygen and nitrogen partial pressures, we plotted the Kellogg diagrams for Ga–O–N by using Gibbs minimization as a function of process parameters (pO_2_ and pN_2_). As seen in Figure 2a, the GaN stability is directly linked to the residual pO_2_ and pN_2_ values, with increasing temperatures causing a limited N_2_ stability region, which shifts to higher values. Therefore, the residual oxygen content, which affects the oxygen activity in the growth chamber, is an extremely important descriptor of GaN stability and synthesizability, even if the total pressure during growth is 1 atm or higher. The pO_2_ values of 10^–25^ atm or lower are for all practical purposes not tunable through external control of pO_2_, however, through the incorporation of well-designed gas mixtures, the pO_2_ is tuned indirectly. To give an example of equilibrium partial pressures of different species during growth under 1 atm total pressure, we demonstrate three cases where nitrogen only (N_2_ + 1 ppm of O_2_), ammonia (NH_3_ + 1 ppm of O_2_), and a carbon-containing mixture with nitrogen gas are used (N_2_ + C + 1 ppm of O_2_). In all cases, we use 1 ppm of oxygen (10^–6^ atm) as the residual content, which should in principle cause the stability to shift toward Ga_2_O_3_ based on the Kellogg diagrams. The rationale behind using 1 ppm of O_2_ is the atmospheric leaks that may exist and also the inlet gases that generally contain such trace oxygen levels. The equilibrium calculations shown in Figure 2b show that although pO_2_ remains too high in the case of N_2_ case alone, the use of ammonia causes a drastic decrease in the pO_2_ level that is below the level required for stability of GaN. This is primarily due to the consumption of oxygen upon ammonia cracking and reactions of the residual oxygen with hydrogen. The same is true and even more significant in the case of incorporating carbon in the system, which drops the pO_2_ even further, primarily due to the Boudouard reaction, which also explains to some extent the efficacy of TEG-a metal–organic precursors^64^ that incorporate carbon during growth. The presence of carbon diminishes the pO_2_ values most effectively, which, in turn, causes GaN stability and inhibits its oxidation. This thermodynamic analysis underscores the importance of nitrogen and gallium precursors in not just the thermodynamic stability of GaN, but also the chemical potential equilibria that emerge during growth, which ultimately dictate the point defect equilibria.

Although the above thermodynamic analysis can guide material synthesis, an explanation regarding the interaction of dopants within the solid solution regime is required. In other words, the above analysis provides a macroscopic picture of phase transitions once each phase is bound to dissociate and demonstrates the changes in the thermodynamic reservoirs based on synthesis conditions that influence point defect stabilities. As the point defects of the GaN phase ultimately dictate the optoelectronic behavior of this material upon doping with Cr, Mn, and O, the *ab initio* study of defect equilibria based on synthesis parameters is of utmost importance. In the following sections, we comprehensively analyze point defect stabilities within the doped GaN solid solution by considering various synthesis conditions.

### 3.2 Intrinsic Defects in GaN

**Figure 3:**
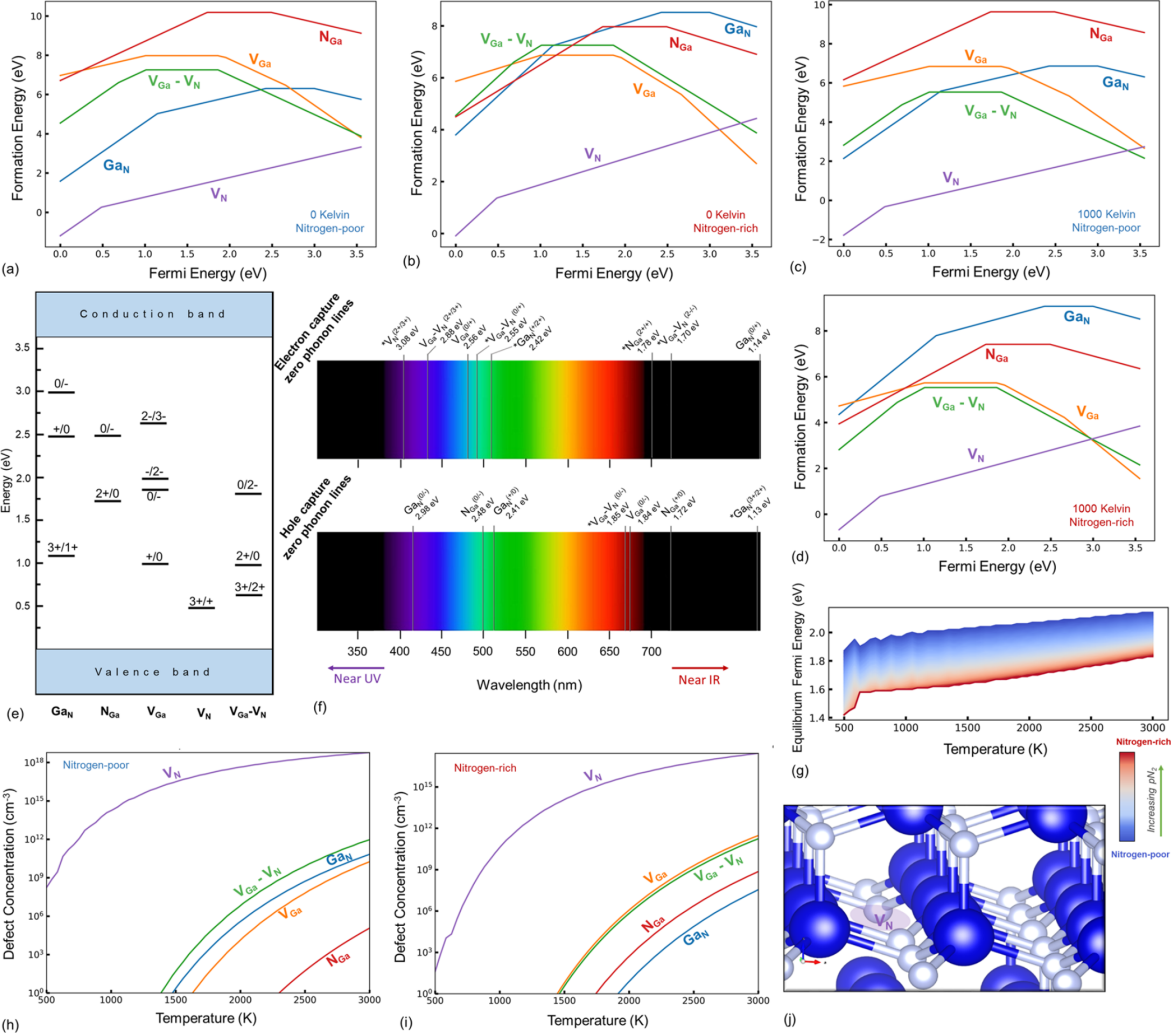
Formation energy versus Fermi energy diagrams for the intrinsic defects at temperatures of 0 K (a, b) and 1000 K (c, d) for nitrogen-rich and -poor conditions, and the charge transitions are shown (e). The associated ZPL lines are illustrated (f) and the changes in equilibrium Fermi energy as a function of temperature (g) shows an increase in the effective band gap under nitrogen-rich growth conditions. The equilibrium defect concentrations as a function of temperature for nitrogen-poor (h) and nitrogen-rich (i) growth show the significance of the V_N_ point defect (i).

The intrinsic defects considered in this study are nitrogen and gallium vacancies (V_Ga_, V_N_, and V_Ga_–V_N_) and the antisite defects of nitrogen and gallium (Ga_N_ and N_Ga_). Based on the calculated formation–Femi energy diagrams shown in Figure 3, the formation of V_N_ is most plausible, which becomes even more dramatic as temperature increases. In addition to the Arrhenius relationship of defect concentrations with respect to temperature (Figures 3h–2i), the formation energy of V_N_ decreases with increasing temperature under any given nitrogen chemical potential (Figure 3a–d). However, under both nitrogen-rich and -poor conditions, the evolution of the V_Ga_–V_N_ defect is the following likely defect. The antisite defects, due to their high formation energy, are the least likely unless in highly minute concentrations. Formation of the V_Ga_ in 2 and 3 charge states is also likely under nitrogen-rich conditions if the equilibrium Fermi energy increases beyond approximately 2.5 eV.

Considering the formation energies in Figure 3, the equilibrium Fermi energy that yields charge neutrality at any temperature can be calculated (Figure 3g). Mainly, due to the contribution of V_N_, the *E*_f_ value tends to approach the conduction band as the nitrogen chemical potential decreases. Due to the reduction of the nitrogen partial pressure (pN_2_) during growth, the *E*_f_ shifts closer to the conduction band, reducing the effective band gap, which enhances the concentration of V_N_. The equilibrium defect concentrations that should be expected (Figure 3h,i) demonstrate the dominance of V_N_ over all other intrinsic defects. However, the concentration of this defect can be reduced by increasing the nitrogen partial pressure or decreasing the temperature during synthesis. Both actions will reduce the equilibrium Fermi energy and thus positively contribute to the bulk dielectric breakdown of GaN. Under nitrogen-poor conditions, V_Ga_–V_N_ defect is the next most likely defect, followed by Ga_N_, while V_Ga_ has a higher equilibrium concentration than V_Ga_–V_N_ under nitrogen-rich conditions.

As can be seen in Figure 3a, the V_N_ defect can be stabilized in 3+ and 1+ charge states. Although a very narrow stability window (∼0.25 eV) of the neutral charge state near CBM has been reported by Lyons et al.,^7^ our results of only 3+ and 1+ charge states being stable agree accurately with the predictions reported by Xie et al.,^23^ Diallo et al.^65^ and Freysoldt et al.^66^ The *E*^3+/+^ charge transition of V_N_ is a prime example of negative-*U* behavior. A defect has negative *U* properties if it can trap two electrons (or holes) with the second bound more strongly than the first. The system can be described as an extrinsic Cooper pair, with the defect providing an environment in which a net attraction can develop between the otherwise coulombically repulsive carriers. The negative *U* behavior is very ubiquitous in many semiconductors hosting point defects, especially binary compounds such as GaN. In fact, there are several defects in our investigations that exhibit this behavior.

Formation of complex defects such as V_Ga_–V_N_ are a subject of both thermodynamic and kinetic constraints. The latter is related to the migration barriers of simple defects, such as V_N_ or V_Ga_. According to the available DFT results,^67,68^ V_Ga_ exhibits a significantly lower migration barrier of 1.9 eV in the n-type range than 4.3 eV of V_N_; therefore, V_Ga_ is a mobile defect and the formation of V_Ga_–V_N_ can be described as trapping a mobile V_Ga_ by V_N_. The defect complex stability, in turn, is related to its dissociation energy approximated by the sum of the migration barrier and the complex binding energy.^67^ Taking the migration barrier of 1.9 eV for V_Ga_ and the calculated binding energy of 3.24 eV for V_Ga_–V_N_, the dissociation energy amounts to 5.14 eV rendering the complex very stable once it is formed.

**Figure 4:**
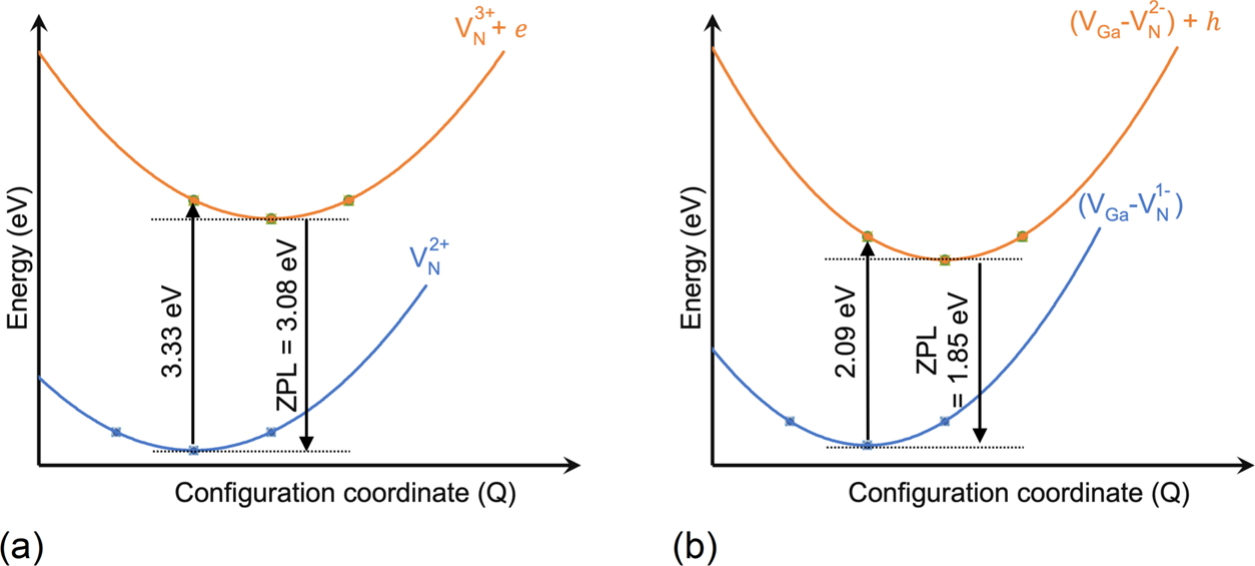
Configuration coordinate diagrams representing (a) electron capture from CBM by V_N_^3+^ and (b) hole capture from the VBM by V_Ga_–V_N_^2–^ defects in GaN.

The equilibrium concentration of V_N_ is orders of magnitude higher than those of the other intrinsic defects studied here. V_N_ is thermodynamically highly favored to form in a 1+ charge state for the majority of equilibrium Fermi energies, as the adiabatic charge transition level *E*^3+/+^ is situated at *E*_VBM_ + 0.48 eV. As a result, hole capture V_N_^2+^ + h → V_N_^3+^ ZPL is in the IR and an electron capture V_N_^3+^ + e → V_N_^2+^ ZPL should be expected at approximately 3.08 eV (see Figure 3f). We plotted the configuration coordinate diagrams (CCD) for two representative transitions, i.e., one for electron capture from CBM by V_N_^3+^, and one for hole capture from VBM by V_Ga_–V_N_^2–^, in order to simplify the interpretation of the calculated ZPLs associated with electron capture and hole capture transitions, respectively, as seen in Figure 4.

A persistent UVL band luminescence broadband at 3 to 3.3 eV is reported in the experimental study of photoluminescence on highly pure GaN by Reshchikov et al.,^69^ with ZPL value expected at 3.26 eV and attributed to an electron capture transition. They also reported a blue luminescence with a ZPL of 2.9 eV, seen only at higher temperatures. The electron capture from CBM transitions of the V_N_ (V_N_^3+^ + e → V_N_^2+^, ZPL = 3.08 eV) and V_Ga_–V_N_ (V_Ga_–V_N_^3+^ + e → V_Ga_–V_N_^2+^, ZPL = 2.88 eV) defects calculated in our study may explain the fundamental atomistic cause of the observations by Reschikov et al.^69^

### 3.3 Oxygen-Doped GaN

**Figure 5:**
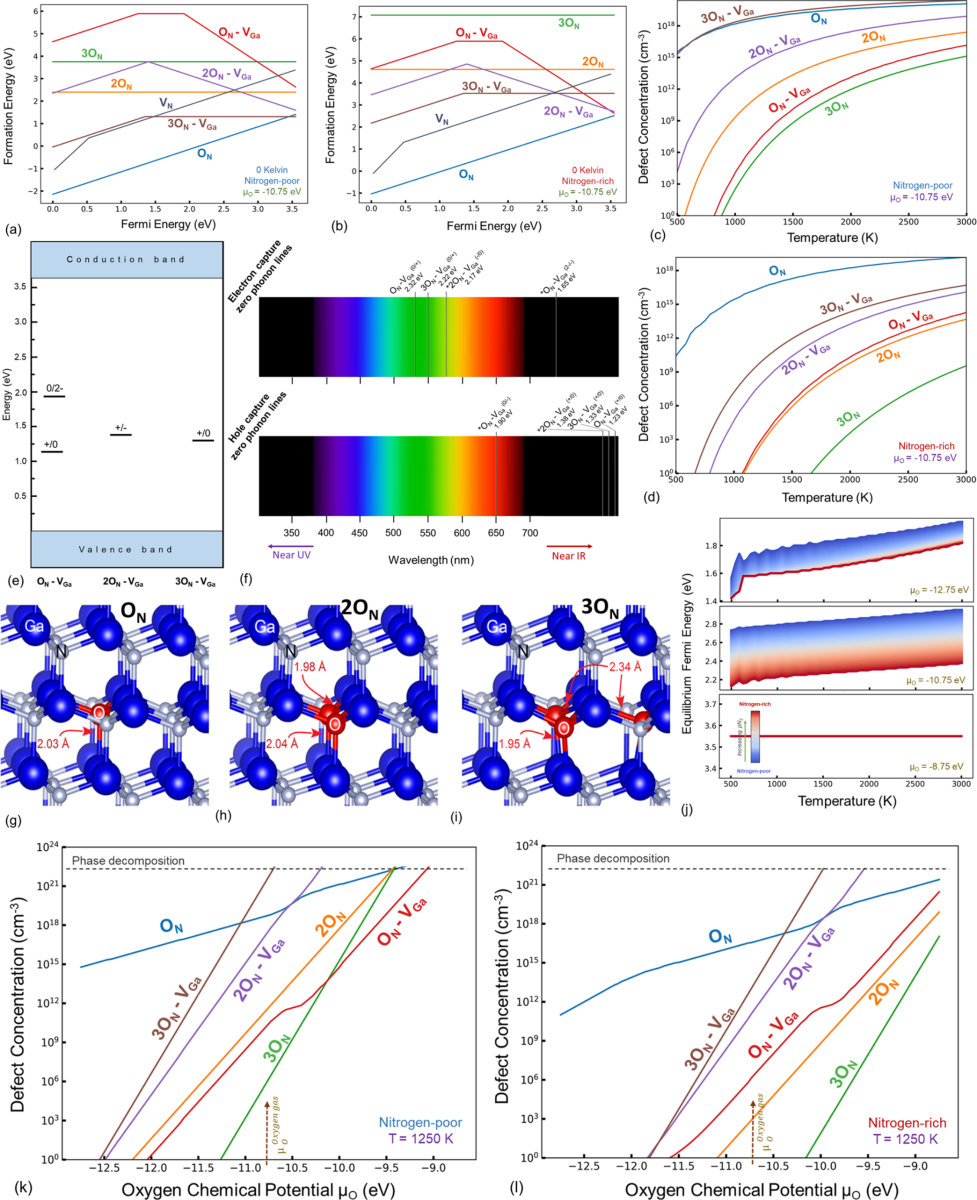
Formation energy versus Fermi energy diagrams for the oxygen and oxygen-intrinsic defects at a temperature of 0 K for nitrogen-rich (a) and -poor (b) conditions demonstrate the significance of ON and 3O_N_–V_Ga_ defects, as seen in the equilibrium concentration plots (c, d) drawn for the oxygen chemical potential referenced to the O_2_ molecule. The charge transitions are shown (e), and the associated ZPL lines are illustrated (f). Ground state structures of *n*O_N_ clusters, where *n* = 1 (g), 2 (h), and 3 (i) considered in this study are shown. The changes in equilibrium Fermi energy as a function of temperature (j) show a significant reduction in the effective band gap under nitrogen-poor growth conditions, and therefore the O_N_ defect can explain the unintentional n-conductivity of GaN. Changing oxygen chemical potential transforms the defect stability diagrams (k, l).

Excessive oxygen activity dissociates GaN and induces a phase transformation to Ga_2_O_3_ (Figures 1g and 2a); however, under reduced oxygen partial pressures, the phase stability of GaN can be obtained. Therefore, oxygen-related defects become inevitable under such conditions, especially when considering process and system designs where the pO_2_ range is hardly lower than 10^–25^ atm. The formation energies of oxygen-related defects depend on the nitrogen partial pressure, and an increase in pN_2_ tends to decrease the equilibrium concentration of the oxygen-related point defects, as anionic nitrogen tends to be further stabilized under higher environmental nitrogen activity, as seen in Figure 5. The O_N_ defect is critical as a dominant oxygen-related defect. O_N_ is only stable in a 1+ charge state regardless of the electron chemical potential, which agrees with the DFT prediction of Xie et al.^70^ However, it becomes probable upon increasing oxygen and nitrogen activity or forming a gallium vacancy adjacent to oxygen-substituted nitrogen sites, and the formation energy of O_N_–V_Ga_ decreases. Upon excessive oxygen chemical potentials, oxygen aggregation around the gallium vacancy increases, forming 3O_N_–V_Ga_, eventually leading to phase decomposition (Figure 5k,l).

**Figure 6:**
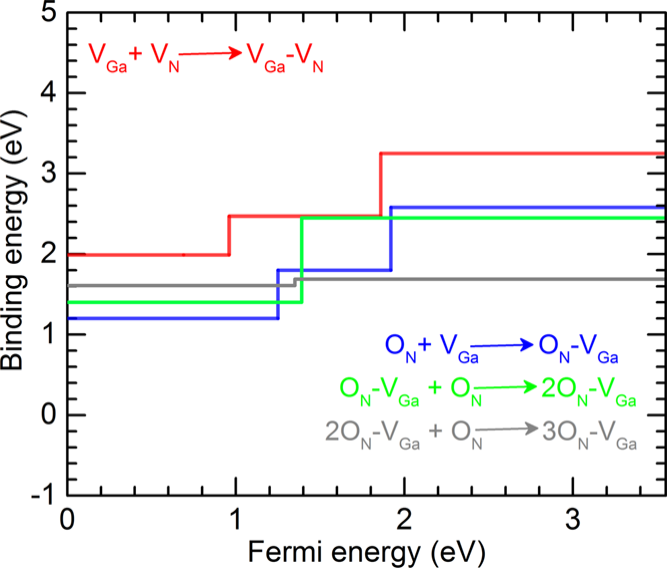
Binding energy of V_Ga_–V_N_ and *n*O_N_–V_Ga_ complexes in GaN as a function of the Fermi level. Charge neutrality condition has been considered.

In fact, the positive binding energy of *n*O_N_–V_Ga_ complexes (see Figure 6) reflects their high stability and tendency of oxygen to cluster with gallium vacancy. Aggregation of oxygen in GaN has also been reported by Michałowski et al.^71^ where most O atoms are agglomerated along pillar-shaped structures. Nevertheless, aggregation of oxygen atoms at adjacent nitrogen sites in the absence of a gallium vacancy is rather unlikely, as seen from the formation energies of 2O_N_ and 3O_N_.

The calculated total concentration of O-related defects ∼2 × 10^16^ cm^–3^ is in excellent agreement with SIMS experimental data for two samples (1.1–3.2) × 10^16^ cm^–3^ measured by Reshchikov et al.^72^ The calculated concentration of O-related defects is further confirmed by Sadovyi et al. for GaN crystals grown by Halide Vapor Phase Epitaxy (HVPE). The experimentally measured value in the bulk of GaN was found to be <10^17^ cm^–3^.^73^ Interestingly, they have concluded that oxygen diffusion in the GaN lattice is anomalously small up to temperatures close to the melting point, which is due to a very high activation barrier. Our calculated binding energies of oxygen to gallium vacancy-related complexes support exceptional stability of *n*O–V_Ga_ defects, acting as a strong trap and thus, inhibiting diffusion of oxygen.

In the case of an O_N_ defect, the oxygen atom substituting nitrogen has one extra electron that is spontaneously ionized, leaving behind a very stable 1+ charge state and one localized hole state in the band gap. The intuition says that 2O_N_ and 3O_N_ should be the most stable in 2+ and 3+ charge states, respectively. It turned out that only the neutral charge state would be considered feasible due to the following reasons. The formation of *n*O_N_ clusters requires bringing *n* isolated O_N_ donors to the nearest neighboring sites (see Figure 5g–i). The nearest oxygen atoms stuck together induce significant Coulombic repulsion, and as a result, the antibonding defect level (where the hole responsible for donor behavior should be localized) is shifted upward, falling into the conduction band, and becoming delocalized. Upon inspecting the Kohn–Sham eigenvalue spectra of *n*O_N_ clusters, we can indeed see the lack of donor state in the band gap. For this reason, the *n*O_N_ anion substitution clusters where *n* > 1, exists in the neutral charge state. We also calculated the 1 and 2 charge states to inspect whether adding electrons can shift the antibonding, O-related levels back in the band gap. The formation energy turned out to be very high for these charge states, deeming them extremely unstable and thus unlikely to form.

Among the oxygen and intrinsic related defects mentioned in this section, only O_N_–V_Ga_, 2O_N_–V_Ga_, and 3O_N_–V_Ga_ can exist in more than one charge state, and their thermodynamic charge transitions and associated electron and hole capture ZPL values are illustrated in Figure 5e,f. The RL band is reported to have a ZPL of 2.36 eV based on the experimental observations by Reshchikov et al.^74^ and a band maximum at 1.80 eV, causing the red luminescence. There have been significant suspicions regarding its origin being the O_N_–V_Ga_ defect.^70^ Our results confirm that the electron capture transition of O_N_–V_Ga_ (O_N_–V_N_^1+^ + e → O_N_–V_N_^0^) has a ZPL value of 2.32 eV, along with a hole capture (O_N_–V_N_^1–^ + h → O_N_–V_N_^0^) ZPL at 1.90 eV, which further strengthens the argument by Xie et al.,^70^ and closely matches experimental findings. However, our calculations also predict a much higher concentration of the 3O_N_–V_Ga_ defect, which has a ZPL of 2.22 eV for the electron capture transition 3O_N_–V_N_^1+^ + e → 3O_N_–V_N_^0^, and this too is almost within the DFT error (±0.1 eV) for the experimental ZPL of the RL band. In contrast, the concentration of this defect is orders of magnitude higher than that of O_N_–V_Ga_. The emergence of the 3O_N_–V_Ga_ defect is dominant upon reduced nitrogen partial pressure or increased oxygen fugacity. Therefore, further comparative analysis and quantum calculations on the optical PL of 3O_N_–V_Ga_ are essential, as this defect may significantly contribute to the RL band of GaN.

The cause of the unintentional n-type conductivity in GaN has also been debated. Early studies had postulated the significance of V_N_ as the primary cause. However, further *ab initio* studies have shown that the V_N_ formation energy and concentration remain limiting factors. The computational results of our study confirm that V_N_ is not a likely candidate, especially when considering that the equilibrium Fermi energy primarily caused by V_N_ remains below *E*_VBM_ + 2 eV, and the Fermi level stabilizes at the gap center (Figure 3g). Therefore, the n-type conductivity is most likely contaminant-related, especially oxygen or carbon-based, as was previously suggested by Neugebauer and Van de Walle.^19,20^ Our study confirms that oxygen can indeed explain the n-type conductivity of GaN crystal. In fact, the carrier concentrations obtained from Van der Pauw–Hall measurements increased an order of magnitude when oxygen was incorporated into the grown layers confirming its shallow donor nature.^75^ When considering Figure 5k,l, an exponential increase in the concentration of O_N_ is seen with increasing oxygen chemical potential or decreasing nitrogen partial pressure. Given that the O_N_ is stable in the 1+ charge state, higher concentrations of this defect shift the equilibrium Fermi level toward the conduction band. As seen in Figure 5j, with increasing oxygen chemical potentials, the equilibrium Fermi energy increases, and this increase can reach a few meV from the conduction band if the oxygen activity becomes sufficiently high enough. Thus, the presence of oxygen causes n-type conductivity and results in the diminishing of the dielectric breakdown voltage.

### 3.4 Cr-Doped GaN

**Figure 7:**
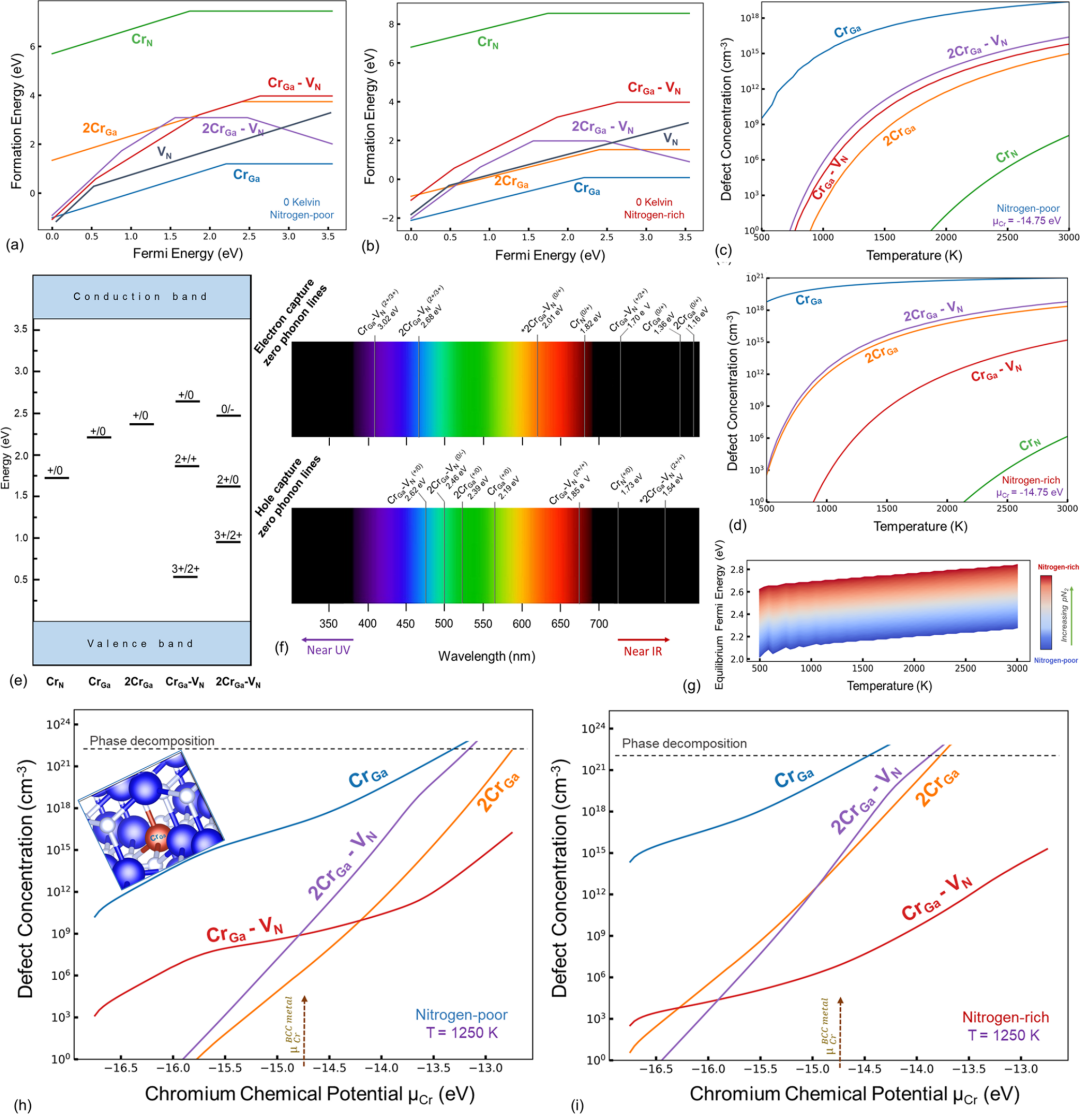
Formation energy versus Fermi energy diagrams for the chromium and chromium-intrinsic defects at a temperature of 0 K for nitrogen-rich (a) and -poor (b) conditions demonstrate the significance of Cr_Ga_ and 2Cr_Ga_–V_N_ defects, as seen in the equilibrium concentration plots (c, d) drawn for the Cr chemical potential referenced to the BCC Cr metal. The charge transitions are shown (e), and the associated ZPL lines are illustrated (f). The changes in equilibrium Fermi energy as a function of temperature (g) show a reduction in the effective band gap under nitrogen-rich growth conditions, primarily due to the dominance of the Cr_Ga_ defect. The Cr chemical potential dependent concentrations for nitrogen-poor (h) and rich (i) conditions are shown as well.

Upon doping of Cr in the GaN structure, substitutional Cr at the Ga site (Cr_Ga_) is a highly favorable point defect (Figure 7a,b). The stability of this defect is significantly higher under nitrogen-rich synthesis conditions (Figure 7c,d). The solubility of Cr in GaN can be extended to the point of complete solubility if the chromium chemical potential exceeds that of Cr metal, which is expected under a plasma state (Figure 7h,i); however, under such conditions, phase decomposition and evolution of the CrN phase are expected, as previously described (Figure 1b). Moreover, under the N_2_-rich conditions, Cr aggregation, as in the formation of 2Cr_Ga_ clusters, is also likely. However, the Cr_N_ defect is unlikely to form in considerable concentrations in both pN_2_-rich and -poor growth conditions, given the high formation energy of this defect compared to the other Cr-related defects considered here. On the other hand, the evolution of 2Cr_Ga_–V_N_ is also highly likely, and this is the second most significant defect after Cr_Ga_ for most synthesis conditions (Figure 7h,i). The formation energy of Cr_Ga_–V_N_ is relatively higher. The charge transitions are listed in Figure 4e for the Cr-related defects,

Among the Cr-related defects, all of them exhibit a triple, double, or single donor behavior. In addition, the 2Cr_Ga_–V_N_ also exhibits an acceptor behavior (exists in the 1– charge state). Optical transitions from the Cr-related defects can be expected from the IR to the visible and UV ranges, demonstrating the possibilities for a wide range of optical devices through Cr-doped GaN (Figure 7f).

However, given the extreme stability of Cr_Ga_, in addition to its internal transition, the adiabatic charge transition ZPL values of approximately 2.19 and 1.36 eV are expected for the hole (Cr_Ga_^0^ + h → Cr_Ga_^1+^) and electron (Cr_Ga_^1+^ + e → Cr_Ga_^0^) capture transitions, respectively. The experimentally observed ZPL of 1.193 eV has been attributed to the Cr_Ga_ by Baur et al.^15^ However, our findings closely resemble this experimental ZPL value with the theoretical (2Cr_Ga_^1+^ + e → 2Cr_Ga_^0^) transition of 2Cr_Ga_. The formation of this defect is unlikely, especially at higher Cr chemical potentials and under highly nitrogen-rich growth conditions.

Moreover, the observation of green coloration in GaN, reported by Zimmermann et al.,^76^ reports a direct correlation between the 1.193 eV ZPL and the Cr content observed by SIMS, further strengthening the Cr-related nature of this peak; however, given the green coloration that they observed only in Cr-rich zones of the crystal points to the importance of studying Cr-related transitions in the proximity of 2 to 2.8 eV as well, which has been only attributed to carbon-related defects (the D-band).^76^ Our results demonstrate that Cr_Ga_, 2Cr_Ga_, and 2Cr_Ga_–V_N_ defects all have hole-capture transitions that can contribute to the D band. The 2Cr_Ga_–V_N_^1–^ + h → 2Cr_Ga_–V_N_^0^ hole capture transition of 2Cr_Ga_–V_N_ has a ZPL of 2.46 eV, where the D band observed by Zimmerman et al. also shows a broad peak. PL studies by Farooq et al.^77^ on Cr-doped GaN further strengthen our argument because they observed an increase in the PL intensity of a defect-related broadband ranging from 550 to 700 nm upon Cr doping that was absent in pure GaN, which was grown from carbon-free precursors.

Similarly, by considering the thermodynamic stabilities of the various Cr-related point defects and their ZPL values, we can explain the green coloration of Cr-doped GaN by considering a Stokes shift of 0.4 eV (based on prior calculations on various other centers)^78^ for the most dominant of the Cr-related defects (Cr_Ga_). This also makes it possible to reproduce the absorption peak of approximately 700 nm, as seen experimentally by Zimmermann et al.^76^

When considering the variations in the equilibrium Fermi energy for the Cr-related defects reported in this section and the intrinsic defects, contrary to the case of intrinsic defects alone, a reduction in *E*_f_ is seen as the nitrogen partial pressure decreases during synthesis. However, adding Cr at any given temperature and nitrogen partial pressure shifts the *E*_f_ values closer to the conduction band, which can negatively impact the dielectric breakdown thresholds while increasing the free charge-carrier concentrations. It is also clear that nitrogen-poor synthesis conditions are favorable to stabilize the Cr_Ga_ defect in the 1+ charge state, as nitrogen-rich conditions tend to stabilize the charge-neutral form of this defect by increasing *E*_f_.

### 3.5 Mn-Doped GaN

**Figure 8:**
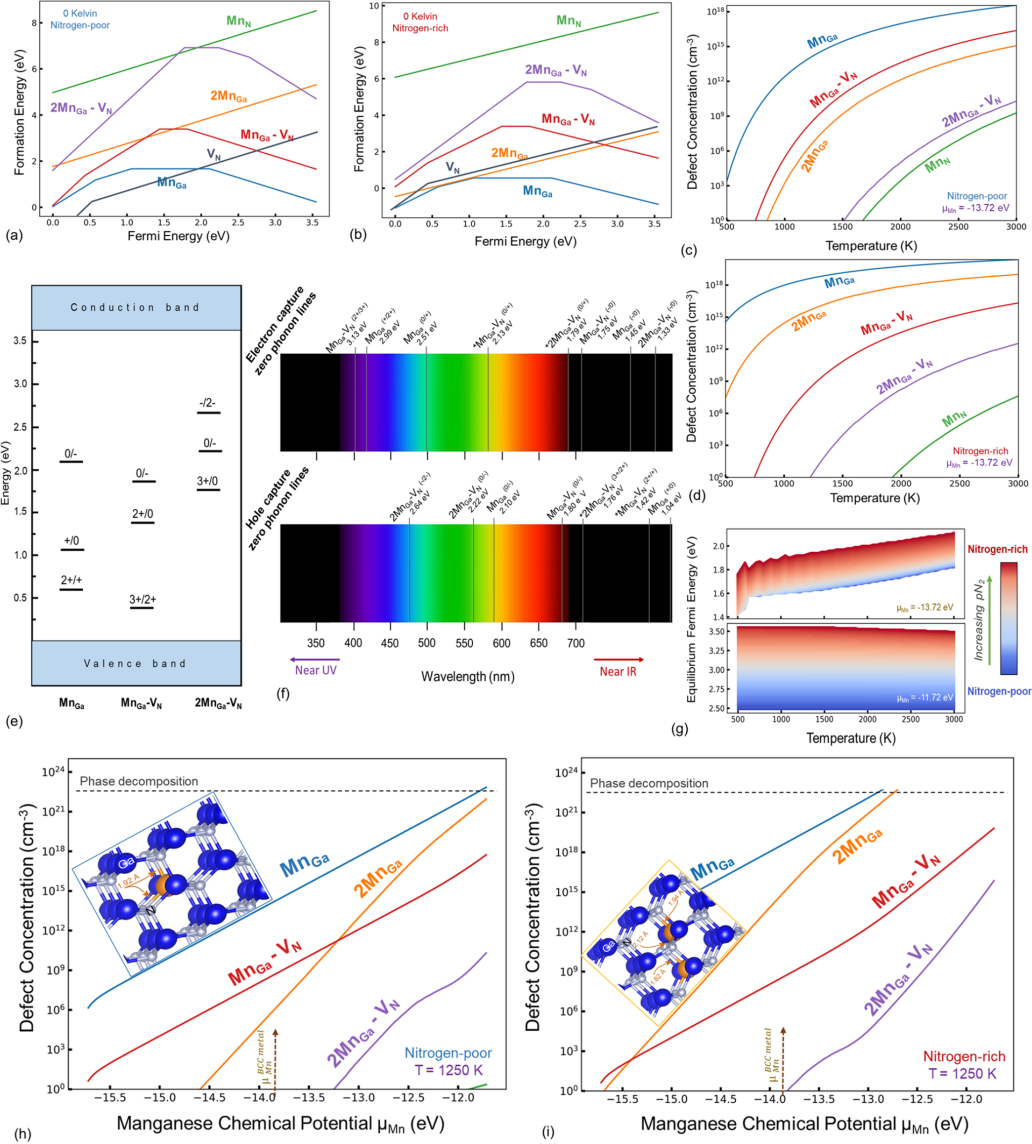
Formation energy versus Fermi energy diagrams for the manganese and manganese-intrinsic defects at the temperature of 0 K for nitrogen-rich (a) and -poor (b) conditions demonstrate the significance of Mn_Ga_, 2Mn_Ga_, and Mn_Ga_–V_N_ defects, as seen in the equilibrium concentration plots (c, d) drawn for the Mn chemical potential referenced to the BCC Mn metal. The charge transitions are shown (e), and the associated ZPL lines are illustrated (f). The changes in equilibrium Fermi energy as a function of temperature (g) show a reduction in the effective band gap under nitrogen-rich growth conditions. An increase in Mn chemical potential (or concentration) tends to change the stable Mn point defects (h, i).

Three of the Mn-related defects are of the utmost importance: Mn_Ga_, 2Mn_Ga_, and Mn_Ga_–V_N_. The Mn_Ga_ is the most dominant Mn-related defect, stable in 2+, 1+, and 1+ charge states in addition to the neutral state. However, Mn has a solubility lower than that of Cr due to its higher formation energy for a wide range of Fermi energies (Figure 8a,b). The Mn_N_ is the least likely of these Mn-related defects, with the 1+ charge state being the only stable form of this defect. For all phase-stable μ_Mn_ values, under both nitrogen-rich and -poor growth conditions, the Mn_Ga_ defect remains the most dominant, with exponentially increasing concentration as the nitrogen chemical potential increases (Figure 8c,d). In nitrogen-rich conditions, the next dominant defect is 2Mn_Ga_; however, decreasing the pN_2_ overtakes the dominance of the Mn_Ga_–V_N_ defect, especially in the 1– charge state. The 2Mn_Ga_, like Mn_N_, is only stable in the 1+ charge state. The charge transition levels and expected ZPL are illustrated in Figure 8e,f. Increasing the Mn concentration in GaN increases the equilibrium Fermi energy, as illustrated in Figure 8g.

As the isolated Mn_Ga_ defects induce significant Coulombic repulsion when stuck next to each other, the defect-related localized states in the band gap shift toward the CB (antibonding states) or VB (bonding states). Thus, the cluster exhibits fewer charge states than does the isolated substitutional defect. As opposed to O_N_, Mn_Ga_ induces a larger number of localized defect states in the band gap due to the presence of 3d orbitals. For the 2Mn_Ga_ case, we find that most of the states are pushed above the conduction or below valence bands, hybridizing with them, and only one shallow donor state remains in the band gap. This, in turn, explains why 2Mn_Ga_ is a shallow donor.

In the case of extreme Mn chemical potentials that may become locally feasible during growth under plasma states, the aggregated Mn defect (2Mn_Ga_) tends to approach the concentration of Mn_Ga_ (Figure 8h,i). Under nitrogen-poor conditions, reduction in Mn chemical potential causes the transformation of 2Mn_Ga_ to the Mn_Ga_–V_N_ defect.

The experimental results of Gelhausen et al.^79^ on the dopant-concentration-dependent changes in the luminescence of GaN are in accordance with our results. Our study confirms their statement regarding the emergence of Mn_Ga_–V_N_ upon heavy doping of Mn at concentrations of approximately 10^20^ cm^–3^ (Figure 8h,i). The results shown in Figure 8f also explain the infrared luminescence of the Mn-doped samples, which may be attributed to the Mn_Ga_ and Mn_Ga_–V_N_ defects. Our findings further indicate that the Mn-related transition, experimentally reported at *E*_VBM_ + 1.8 eV by Graf et al.^80^ and Wolos et al.,^81^ is most likely caused by the Mn_Ga_–V_N_^1–^ + h → Mn_Ga_–V_N_^0^ hole capture transition of the Mn_Ga_–V_N_ defect and not by Mn_Ga_. The strong blue luminescence reported in Mn-implanted GaN with a peak at approximately 3 eV and a level at *E*_VBM_ + 0.43 eV, reported by Polyakov et al.,^82^ can be explained by the Mn_Ga_^2+^ + e → Mn_Ga_^1+^ electron capture transition of Mn_Ga_. The Mn_Ga_–V_N_^3+^ + e → Mn_Ga_–V_N_^2+^ electron capture transition of the Mn_Ga_–V_N_ defect contributes to the near UV luminescence as well. Similarly, the *E*_VBM_ + 1.42 eV deep level experimentally seen by Korotkov et al.^83^ is attributed to the *E*^2+/+^ charge transition of the Mn_Ga_–V_N_ defect.

We also suspect possible contributions from Mn_Ga_ in the broad high-energy absorption band (centered at 2.7 eV). Therefore, further investigation of the configuration coordinate diagrams for the dominant defects stated here will prove helpful for providing a conclusive identification of the optical transitions and assigning them to their rightful atomistic origin.

### 3.6 Cr- and O-Codoped GaN

**Figure 9:**
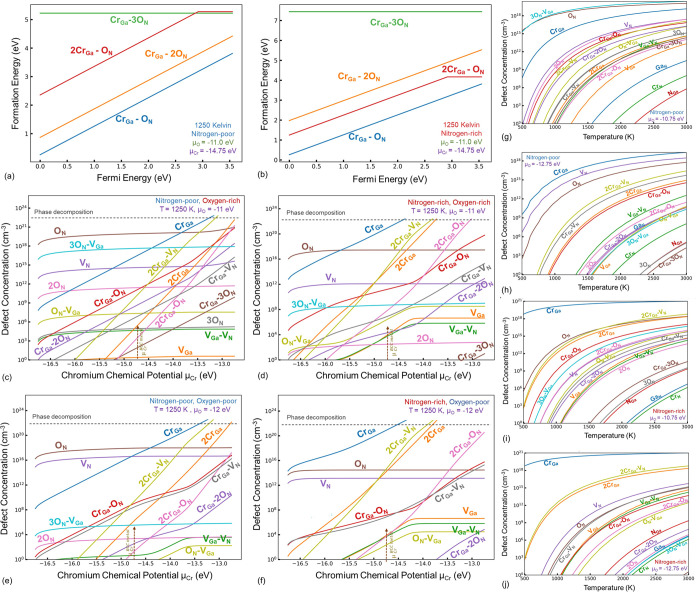
Formation energy versus Fermi energy diagrams for the chromium and oxygen-related defect complexes at a temperature of 1250 K for nitrogen-rich (a) and -poor (b) conditions demonstrate their relatively high formation energies and insignificance as seen in the equilibrium concentration plots for nitrogen/oxygen-rich and -poor growth conditions (c–f). The temperature-dependent equilibrium concentrations are also shown (g–h) for the Cr chemical potential referenced to the Cr metal for the nitrogen-rich and -poor growth conditions, referenced to two oxygen chemical potentials of extremely low −12.75 eV and high value of −10.75 eV (O_2_ molecule).

Considering the case of Cr-doped GaN again, but with the assumption that there exists some oxygen codoping as well, a more comprehensive analysis can be done closer to the experimental growth conditions, as the complete elimination of oxygen during growth is rather difficult based on our CALPHAD analysis given in Section 3.1. We considered Cr–O complex defects, shown in Figure 9a,b. Among these defects, Cr_Ga_–O_N_ and Cr_Ga_–2O_N_ are only stable in the 1+ charge state, and Cr_Ga_–3O_N_ is only stable in the neutral charge state. The 2Cr_Ga_–O_N_ has an *E*^+/0^ charge transition at *E*_VBM_ + 2.90 eV. The defect concentrations depend on the chemical potentials of oxygen, nitrogen, and chromium. The chemical potentials depend on the growth conditions, temperature, dopant precursors, and process parameters in large. Therefore, an experimentalist growing GaN indirectly changes the chemical potentials by changing the growth parameters. For example, plasma-assisted growth is bound to significantly increase the dopant chemical potential (dopant activity), affecting the dopant concentrations and the dopant-induced defect. Therefore, in Figure 9, we provide defect concentrations as a function of Cr and O chemical potentials for nitrogen-rich and -poor growth conditions (Figure 9c–f).

The Cr–O complexes studied here do not exhibit significant concentrations, and the dominant Cr-related defect remains as Cr_Ga_. The dominance of O_N_ over V_N_ for a wide range of oxygen chemical potentials further justifies the O_N_-related n-type conductivity of GaN rather than V_N_. Moreover, the only possible solution to eliminating the O_N_ defect is increasing the nitrogen partial pressure and reducing residual oxygen. Cr codoping is also found to increase the concentration of oxygen-related defects slightly. However, under extremely high oxygen chemical potentials, the 3O_N_–V_Ga_ defect tends to surpass the concentration of the O_N_, as seen in Figure 9g.

### 3.7 Mn- and O-Codoped GaN

**Figure 10:**
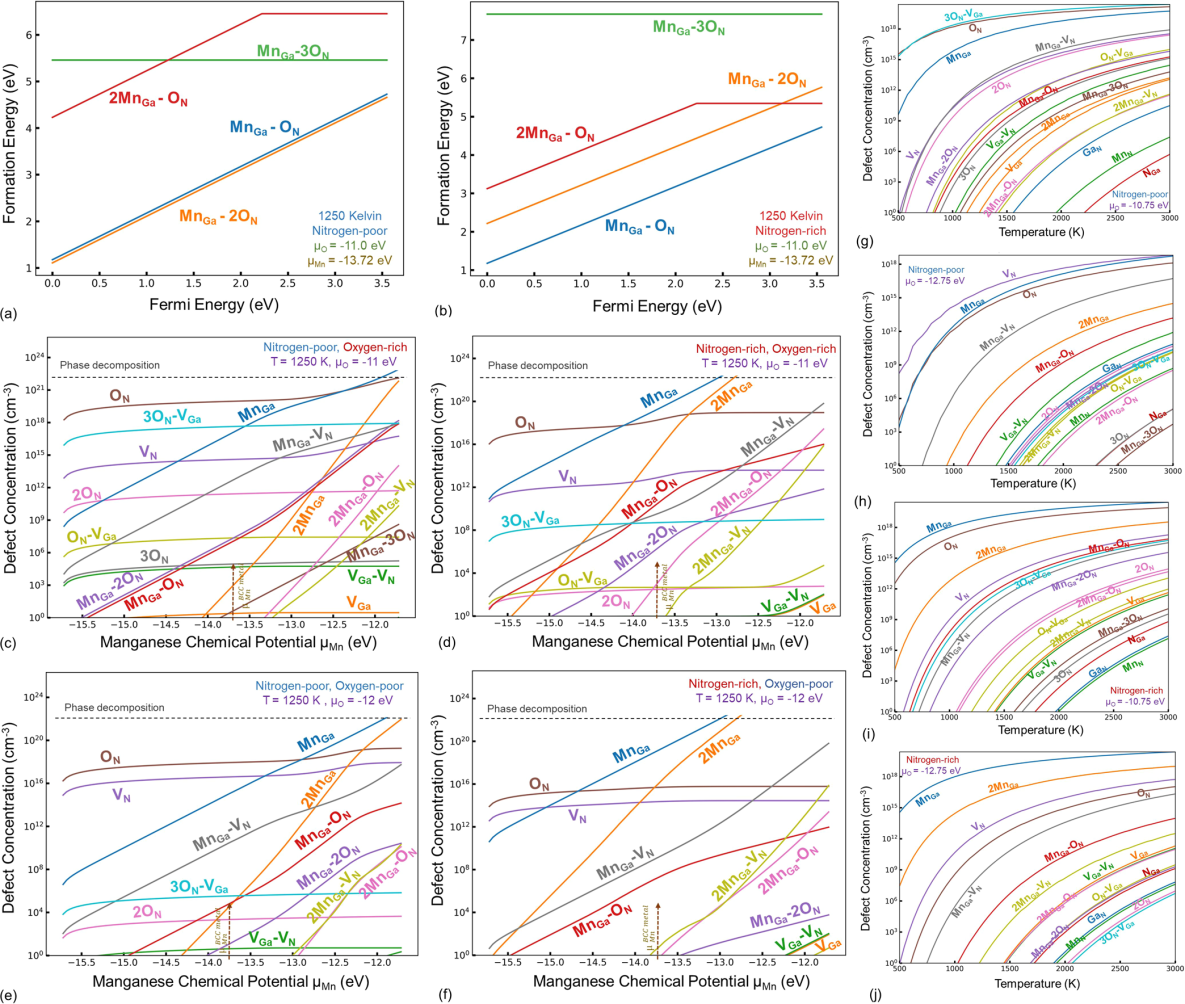
Formation energy versus Fermi energy diagrams for the manganese- and oxygen-related defect complexes at a temperature of 1250 K for nitrogen-rich (a) and -poor (b) conditions demonstrate their relatively high formation energies and insignificance as seen in the equilibrium concentration plots for nitrogen/oxygen-rich and -poor growth conditions (c–f). The temperature-dependent equilibrium concentrations are also shown (g–h) for the Mn chemical potential referenced to the Mn metal for the nitrogen-rich and -poor growth conditions, referenced to two oxygen chemical potentials of an extremely low value of −12.75 eV and a high value of −10.75 eV (O_2_ molecule). The presence of O-related defects tends to stabilize Mn_Ga_–V_N_.

The manganese–oxygen defect complexes we have considered are given in Figure 10a. Among them, the Mn_Ga_–3O_N_ is stable only in the charge-neutral form. The Mn_Ga_–O_N_ and Mn_Ga_–2O_N_ defects are stable in the 1+ charge state, whereas the 2Mn_Ga_–O_N_ defect has an *E*^+/0^ charge transition level at *E*_VBM_ + 2.21 eV. Similar to the Cr case, the Mn–O defect complexes do not demonstrate a significant concentration under any synthesis condition (Figure 10c–f).

Similar to the case of Cr–O codoping, Mn–O codoping tends to increase the equilibrium concentrations of oxygen-related defects, especially under nitrogen-poor growth conditions. The significance of the Mn_Ga_–V_N_ defect was emphasized in Section 3.5, and a series of experimental observations point toward the electronic structure of this defect based on our calculations.

The equilibrium concentration of Mn_Ga_ remained dominant, unless the Mn chemical potential became excruciatingly high. However, when the impact of oxygen codoping is considered, although the oxygen-related complexes do not play a significant role, oxygen changes the equilibrium Fermi energy. Therefore, the defect stabilities under nitrogen-poor growth conditions, in the presence of oxygen signify the exponential increase in the concentration of the Mn_Ga_–V_N_ defect (Figure 10c,e), which is consistent with the low-pressure synthesis conditions of GaN reported in a plethora of experimental studies, where residual oxygen tends to be inevitable, further explaining the experimental observations, and strengthening the conclusion regarding the Mn_Ga_–V_N_ atomistic nature of a range of optical phenomenon.

Our findings demonstrate that increasing the concentration of the oxygen-related defects tends to increase the concentration of the Mn_Ga_–V_N_ defect in GaN, further causing its dominance over other Mn-related defects. However, among the oxygen-related defects, the stabilities remain the same as those described in Section 3.3, and the highest concentration centers remain O_N_ under nitrogen-rich and 3O_N_–V_Ga_ defects under extremely oxygen-rich conditions. In other words, oxygen-aggregated defects emerge with increasing oxygen chemical potentials.

## 4 Summary and Conclusions

We have conducted a comprehensive defect thermodynamic study of oxygen, chromium, manganese doped, and codoped GaN. Our results provide a comprehensive thermodynamic recipe for designing growth conditions of Cr, Mn, and O-doped GaN crystals in order to target a specific defect of interest. We have calculated the process-dependent dominant defects and their concentrations, which is crucial for fabricating high-quality single photon emitters in GaN such as positively charged Cr_Ga_ and diluted magnetic semiconductors based on Mn in GaN, featuring high intrinsic magnetic moment and stability. Some of our findings can be summarized as follows.

- Among the intrinsic defects, V_N_ in the 1+ charge state possesses the highest equilibrium concentration.
- O_N_ is a highly dominant oxygen-related defect that is the most substantial candidate for unintentional n-type conductivity in GaN. The concentration of O_N_ increases under nitrogen-poor growth conditions. Incorporating Mn or Cr also increases the concentration of oxygen-related defects. This defect can be suppressed through growth under high pN_2_ conditions.
- Among the Cr-related defects, Cr_Ga_ is particularly dominant, with increasing concentration under nitrogen-rich growth conditions. Cr_Ga_ possesses a charge *E*^+/0^ transition level at *E*_VBM_ + 2.19 eV.
- Cr_Ga_ in 1+ charge state is stabilized in the presence of oxygen, or nitrogen-poor growth conditions.
- Mn doping causes dominant defects that change based on the growth process parameters. Mn_Ga_ is dominant in a wide range of Mn chemical potentials. The charge transition levels of Mn_Ga_ are situated at 0.56, 1.04, and 2.10 eV above the valence band maximum.
- Heavier doping or increased Mn chemical potentials signify the dominance of 2Mn_Ga_, followed by Mn_Ga_–V_N_.
- The concentration of Mn-related defects increases under nitrogen-rich growth conditions. However, incorporating oxygen in GaN tends to change the dominant aggregated Mn-related defects into Mn_Ga_–V_N_.
- Controlling the residual oxygen content in the growth chamber is essential for increasing the dielectric breakdown voltage.

## Data Availability

We will be happy to share the raw data and the code developed for this study upon a reasonable request.
